# Protein and miRNA profile of circulating extracellular vesicles in patients with primary sclerosing cholangitis

**DOI:** 10.1038/s41598-022-06809-0

**Published:** 2022-02-22

**Authors:** Davide Povero, Masahiko Tameda, Akiko Eguchi, Wenhua Ren, Jihoon Kim, Robert Myers, Zachary D. Goodman, Stephen A. Harrison, Arun J. Sanyal, Jaime Bosch, Lucila Ohno-Machado, Ariel E. Feldstein

**Affiliations:** 1 Department of Biochemistry and Molecular Biology, Mayo Clinic, Rochester, MN USA; 2 Division of Pediatric Gastroenterology, Hepatology, and Nutrition, Department of Pediatrics, University of California San Diego, 3020 Children’s Way, MC 5030, San Diego, CA 92103-8450 USA; 3 Genomics and Microarray Core, University of Colorado Denver, Aurora, CO USA; 4 Department of Biomedical Informatics, University of California San Diego, La Jolla, CA USA; 5 Gilead Sciences, Inc., Foster City, CA USA; 6 Inova Fairfax Hospital, Falls Church, VA USA; 7 Pinnacle Clinical Research, San Antonio, TX USA; 8 Virginia Commonwealth University, Richmond, VA USA; 9 Department of Visceral Surgery and Medicine and Department for Biomedical Research, Inselspital, University of Bern, Bern, Switzerland; 10 Institut d’Investigacions Biomèdiques August Pi I Sunyer (IDIBAPS)-CIBERehd, University of Barcelona, Barcelona, Spain

**Keywords:** Diagnostic markers, Predictive markers, Prognostic markers, Primary sclerosing cholangitis

## Abstract

Primary sclerosing cholangitis (PSC) is an idiopathic and heterogenous cholestatic liver disease characterized by chronic inflammation and fibrosis of the biliary tree. Currently, no effective therapies are available for this condition, whose incidence is rising. At present, specificity and sensitivity of current serum markers used to diagnose PSC are limited and often unreliable. In this study, we characterize circulating extracellular vesicles and provide supporting data on their potential use as novel surrogate biomarkers for PSC. EVs are membrane surrounded structures, 100–1000 nm in size, released by cells under various conditions and which carry a variety of bioactive molecules, including small non-coding RNAs, lipids and proteins. In recent years, a large body of evidence has pointed to diagnostic implications of EVs and relative cargo in various human diseases. We isolated EVs from serum of well-characterized patients with PSC or control subjects by differential centrifugation and size-exclusion chromatography. A complete characterization identified elevated levels of circulating EVs in PSC patients compared to healthy control subjects (2000 vs. 500 Calcein-FITC + EVs/μL). Tissue and cell specificity of circulating EVs was assessed by identification of liver-specific markers and cholangiocyte marker CK-19. Further molecular characterization identified 282 proteins that were differentially regulated in PSC-derived compared to healthy control-EVs. Among those, IL-13Ra1 was the most significantly and differentially expressed protein in PSC-derived EVs and correlated with the degree of liver fibrosis. In addition to protein profiling, we performed a miRNA-sequencing analysis which identified 11 among established, liver-specific (e.g., miR-122 and miR-192) and novel miRNAs. One of the newly identified miRNAs, miR-4645-3p, was significantly up-regulated fourfold in PSC-derived EVs compared to circulating EVs isolated from healthy controls. This study provides supporting evidence of the potential role of circulating EVs and associated protein and miRNA cargo as surrogate noninvasive and reliable biomarker for PSC.

## Introduction

Primary sclerosing cholangitis (PSC) is a rare chronic liver disease whose cause remains unknown yet incidence is increasing^1^. The pathogenesis of PSC is characterized by chronic inflammation and fibrosis of the intra- and extra-hepatic biliary tree. These conditions lead to hepatic fibrosis, cholestasis and potentially to liver cirrhosis, end-stage liver disease frequently requiring liver transplantation^2^. Notably, PSC is a risk factor for the development of cholangiocarcinoma, the second most common liver cancer. At present, there are no standardized and widely accepted diagnostic or prognostic criteria for PSC and common serum markers of liver functionality—such as alkaline phosphatase and aminotransferase—reflect cholestasis but not its cause. At present, all randomized controlled trials of compounds designed to prevent PSC progression have generated negative outcomes and no therapeutic options besides liver transplantation are currently available for PSC patients. Recently, a large body of evidence has demonstrated that during PSC progression, cholangiocytes undergo senescence and release EVs, which induce proliferation, progression and migration of normal and malignant human cholangiocytes^3^. Another elegant report has identified not only elevated levels of EVs circulating in serum in patients with PSC compared to control subjects, but also identified EV-based proteomic signatures that showed potential for applications as novel surrogate biomarkers^4^. Extracellular vesicles are membrane surrounded biological structures with a size range between 100–1000 nm, released by potentially any type of cell as a consequence of cell death, differentiation or normal cell functions^5–7^. Biologically and diagnostically, EVs are very attractive because they shuttle a variety of bioactive molecules, including proteins, short and long non-coding RNAs and lipids, which may provide unique disease-specific signature for novel biomarkers^8–10^. In our previous study, we identified disease-specific protein signatures in circulating EVs of human subjects with cirrhotic and non-cirrhotic nonalcoholic steatohepatitis (NASH)^11^. In this study we characterized large EVs isolated from human subjects with PSC and non-disease healthy control subjects. We identified molecular traits in EVs that may provide additional evidence on the role of EVs as potential surrogate biomarkers for PSC (Supplementary Figure 1).

## Materials and methods

### Patient samples

Baseline serum samples were collected from Primary Sclerosing Cholangitis (PSC) subjects (n = 25), who participated in phase 2 randomized trials evaluating the safety and efficacy of Simtuzumab versus placebo (Clinicaltrials.gov NCT01672866 and NCT01672879) prior to the trial initiation^12^. As controls, serum from 25 healthy control subjects who participated in pharmacokinetics (PK) and phase-I trials were evaluated. Subjects 18–70 years old with chronic cholestatic liver disease due to PSC as confirmed with liver biopsy and MRCP and that had AST and ALT ≤ 10 × the upper limit of normal (ULN) were included in the study. Subjects with ulcerative colitis (UC), a partial Mayo score > 4, partial Mayo bleeding score > 1, use of oral corticosteroids and inhibitors of TNF-α or α4β7 integrin, subjects with secondary sclerosing cholangitis, primary biliary cholangitis, viral hepatitis or alcohol-associated liver disease and hepatic decompensation were excluded from this study. Additional details on exclusion and inclusion criteria are provided in our previously published report^12^. Demographics and baseline characteristics of the study population are summarized in Table 1. All studies were carried out in accordance with relevant guidelines and regulations and all the protocols were approved by the Institutional Review Board of the University of California San Diego, prior to study initiation. Informed consent was obtained from all the subjects included in this study.

**Table 1:** Baseline characteristics of the study population.

	Healthy controls (n = 25)	PSC (n = 25)
**Demographics**		
Age, years	54 (22–70)	42 (21–64)
Male	28% (7)	72% (18)
BMI (kg/m^2^)	24 (19–28)	27.3 (18.5–45.6)
**Live biochemistry**		
ALT (U/L)	–	101 (19–266)
**Fibrosis markers**		
ISHAK	–	4.6 (3–6)
MQC	–	7.2 (1.8–25.3)
Baseline Lysyl Oxidase Like-2 (pg/mL)	–	214.6 (59–859)

All data are presented as the median (interquartile range) or percentage (n). Our PSC study cohort had a lower prevalence of IBD than the broader PSC population and all patients with IBD had quiescent disease. *BMI* body mass index, *MQC* morphometric quantitative collagen, *ALT* alanine aminotransferase.

### Study assessments

Liver histologic assessments were performed as previously described^12^.

### Isolation and characterization of circulating extracellular vesicles

EV isolation, identification, quantitation, size determination and TEM-based morphological characterization were performed according to MISEV 2018 guidelines and as previously reported^11^. Briefly, a volume of 500 μL of serum was centrifuged with an Eppendorf 5415D top bench centrifuge (Eppendorf, Germany) at 400×*g* for 10 min at 4 °C. Supernatants were transferred into new tubes and pellets discarded. Supernatants were further centrifuged at 2500×*g* for 15 min at 4 °C and supernatants were transferred into different tubes while pellets were discarded. A volume of 125 μL of cell and cell debris-free supernatant was added to a qEVoriginal/70 nm SEC column (iZON Science, Medford, MA, USA) for size-exclusion chromatography (SEC), according to the manufacturer’s instructions. Fractions 7 to 9 (equal to 1.5 mL total volume), containing most of EVs, were collected and combined in one tube. Circulating EV identification was performed by EV specific Calcein AM labeling (BD Biosciences, San Jose, CA, USA) and Calcein + -EVs were quantified by BD LSRII Flow Cytometer System (BD Biosciences, San Jose, CA, USA). Data was analyzed using FlowJo software (TreeStar Inc., Ashland, OR, USA). Identification of hepatocyte-derived circulating EVs, lightning-link conjugation kit (Novus Biologicals, Littleton, CO, USA) was used to generate phycoerythrin (PE)-conjugated anti-human antibodies for ASGPR1 (GeneTex, cat. n. GTX122674, San Diego, CA, USA) and SLC27A5 (Thermo-Fisher, cat. n. MA5-17175, Rockford, IL, USA). Expression levels of SLC27A5 and ASGPR1 in circulating EVs were normalized to the total number of EVs in each group.

### Protein isolation and western blotting

Purified circulating EV proteins were extracted through five freeze/thaw cycles, which result in greater concentration of extracts and enrichment of low abundant proteins. Protein concentration was determined by micro-BCA protein assay kit (Thermo Scientific, cat. n. 23235, Waltham, MA, USA), according to manufacturer instructions and as previously detailed^11^. Briefly, approximatively 10 µg of EV protein lysates were solubilized in Laemli buffer, resolved by a 4–20% Criterion Tris–HCl gel electrophoresis system (Biorad, Hercules, CA, USA) and transferred to a 0.2 µm nitrocellulose membrane (Biorad, Hercules, CA, USA). Primary rabbit polyclonal antibodies against Alix, CD61 (1:1000; Genetex, Irvine, CA, USA) and CK-19 (1:500; Santa Cruz Biotechnologies, Santa Cruz, CA, USA) were incubated overnight at 4 °C. Proteins were visualized by Supersignal West Pico chemiluminescence substrate (Pierce Biotechnology, Rockford, IL, USA).

### SOMAscan proteomics array and data analysis

The EV proteomics profile was determined by SOMAscan protein array (SomaLogic, Boulder, CO, USA) in the top seven healthy controls and PSC samples for number of circulating EVs to increase assay specificity and sensitivity. SOMAscan protein assay included 1345 unique proteins.

Samples were submitted to the Genomic and Microarray Core Facility at University of Colorado. Based on SOMAmer™ (Slow Off rate Modified Aptamer) technology, the 1345 proteins were quantified simultaneously in all samples, as previously described^11^. Briefly, pre-normalized SOMAscan expression signal data were read in by R readat package^13^. The dataset analysis of the differentially expressed proteins was performed by an independent statistician in the Genomics and Microarray Core, University of Colorado Denver (Aurora, CO, USA), as previously described^14^. Briefly, log2 transformed SOMAscan proteomic data was analyzed with linear regression model fit followed by Empirical Bayes statistical tests using limma package for differential gene expression^15^. Unsupervised complete linkage clustering of the samples was performed on the top 33 most significantly changed (based on adjusted p-values) proteins in healthy controls vs PSC. The normalized log2 expression values of each sample were used for the clustering analysis and the significantly up- and down-regulated proteins displayed in the heatmap and bar plots have an adjusted p-value of 0.05 or 0.005.

### Isolation of EV-encapsulated miRNAs

Encapsulated miRNAs were extracted from circulating EVs using miRNase (Qiagen, Germantown, MD, USA), according to the manufacturer’s instructions. Briefly, templates were made from 10 ng of total RNA using an TaqMan advanced microRNA cDNA synthesis kit (Life Technologies). Real-time PCR quantification for miRNA expression was performed using a TaqMan miRNA expression assay from Life Technologies. Cycle quantification (Cq) value was converted to relative number using power formulation.

### Sequencing of EV-miRNAs and data analysis

The miRNA sequencing work was carried out in the University of California San Diego (UCSD) IGM Genomics Center. Quality and quantity of purified total RNA from purified circulating EVs was assessed using an Agilent Tapestation and a NanoDrop ND-1000, respectively. Libraries were generated from 100 ng of total RNA, using the TruSeq SmallRNA Sample Prep Kit (Illumina, San Diego, CA, USA) following manufacturer’s instructions. Library quality was assessed using a High Sensitivity DNA kit (Agilent, Santa Clara, CA). Libraries were multiplexed and sequenced with 75 base pair (bp) single end^12^ reads on an Illumina HiSeq4000. The raw sequencing reads in FASTQ format went through preprocessing first to trim 3′ adapter and next to keep only reads whose length is between 17 and 27 bases. Using bowtie, the survived reads were aligned to reference resources such as mouse genome, miRNA precursor sequence, and non-coding RNAs, allowing only up to two mismatches. Only the reads mapped to both genome and precursor, but not to small RNAs other than miRNA were selected for expression level quantification. Then miRNA expression counts were quantified, normalized, calculated for fold-change between the treated and the control groups, and tested for significance of differential expression using negative-binomial model^16^. The top 11 significantly (raw p-value less than 0.05) up- and down-regulated miRs were chosen for volcano plot, clustering and heatmap analysis and pathway enrichment analysis. For each miRNA, a set of target genes was created with a threshold of prediction score 0.7 against micro CDS target prediction database using DIANA TOOLS^17^. The union set comprising all 20 sets of target gene was mapped to Kyoto Encyclopedia of Genes and Genomes (KEGG) pathways^18–20^ to derive significantly enriched pathways using Fisher’s exact test. A volcano plot was generated and differentially expressed EV-miRNAs in PSC vs. healthy control samples are reported as log2Foldchange, using p < 0.05. Two heatmaps, miRNA-by-sample and miRNA-by-pathway, were drawn with Heatmapper and DIANA-miRPath, respectively.

### Statistical analysis

Data is expressed as the mean ± SD unless otherwise indicated. Differences between three or more groups were compared by a nonparametric Kruskal–Wallis ANOVA test. If a significant effect was detected, post-hoc pair-wise comparisons were performed using Mann–Whitney tests with Bonferroni correction. Differences between two groups were compared by a two-tailed Student’s t-test if data had a normal distribution or a Mann–Whitney test if the data deviated from the normal distribution. Differences were considered to be statistically significant at p < 0.05. All statistical analyses were performed using GraphPad Prism 4.0c (La Jolla, CA, USA) or R v3.0.2 (http://www.r-project.org).

## Results

### Circulating EVs in subjects with primary sclerosis cholangitis

**Figure 1:**
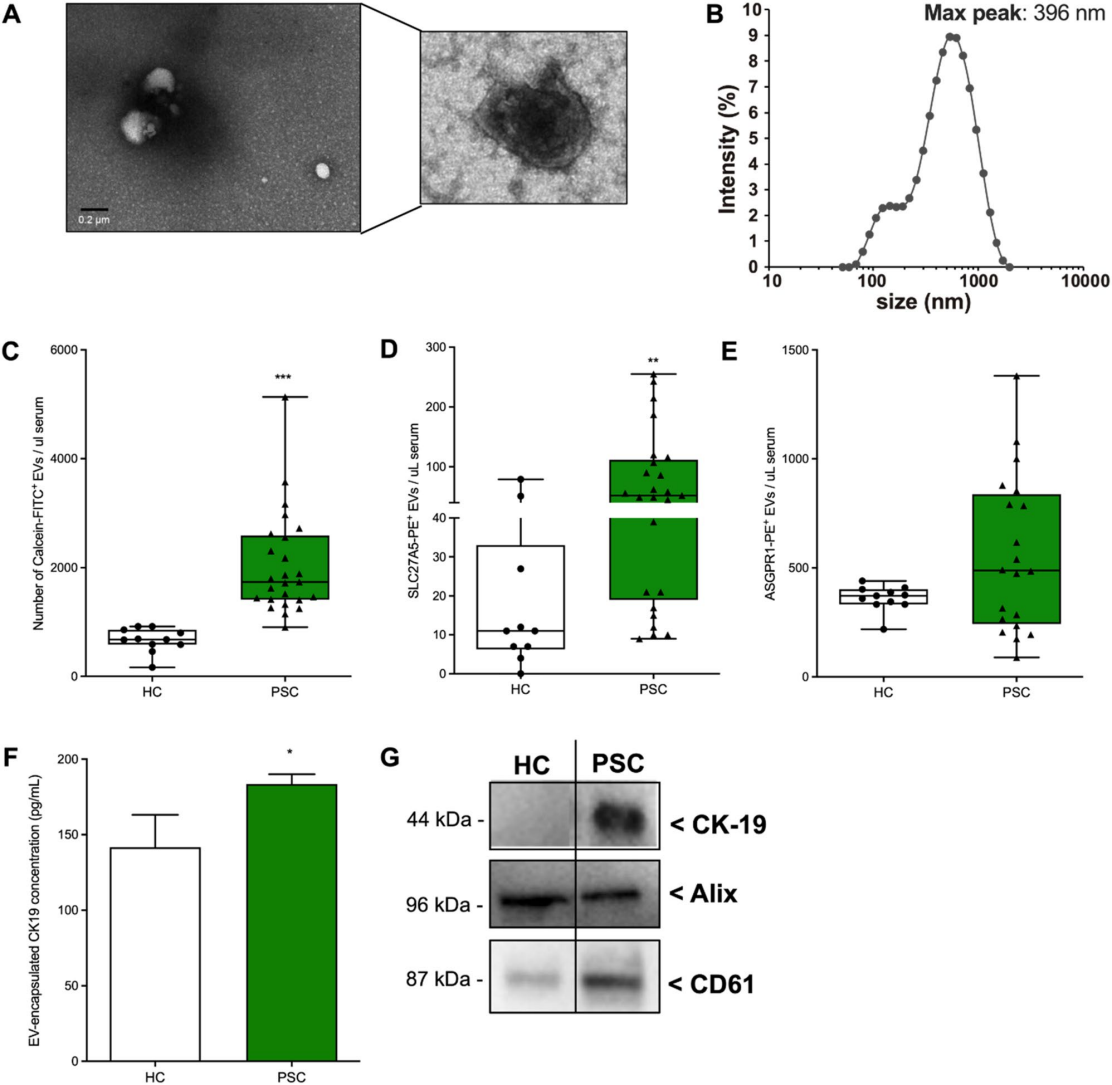
Identification and characterization of circulating EVs in PSC patients. (**A**) Representative scanning electron microscopy images of circulating EVs isolated from PSC patients. (**B**) Dynamic light scattering analysis of the size^31^ of circulating PSC-derived circulating EVs (max peak: 396 nm). (**C**) Flow cytometry analysis of calcein/FITC + circulating EVs detected in healthy controls (n = 11) and PSC patients (n = 25). (**D**,**E**) Flow cytometry analysis of circulating EVs positive for hepatocyte markers SLC27A5 or ASGPR1. (**F**) Flow cytometry analysis of circulating EVs positive for cholangiocytes marker CK-19. (**G**) Western blot analysis of EV marker Alix and CD61 and cholangiocyte marker CK19 in healthy control and PSC-derived circulating EV protein lysates (samples derive from the same experiment and blots were processed in parallel). Values represent mean ± SD. Kruskal–Wallis test with post-hoc Mann–Whitney test and Bonferroni correction were used for statistical analysis.

Circulating extracellular vesicles have emerged as novel and potential liquid biopsies for several pathological conditions^10,21–23^. Cholangiocyte undergoing senescence during PSC, release large amounts of extracellular vesicles^3^. We firstly characterized circulating EVs isolated from healthy controls and PSC samples by flow cytometry, dynamic light scattering and electron microscopy. Analysis of PSC-EV morphology by electron microscopy, identified EVs as typical membrane-surrounded cup-shaped structures (Fig. 1A). Determination of EV size by dynamic light scattering, identified a heterogeneous population with a diameter ranging from 200 to 1000 nm and a maximum peak at around 400 nm (Fig. 1B). This indicate that our EV isolation strategy yielded preferentially large vesicles or microparticles. No difference in EV size was found between healthy control and PSC (data not shown). Flow cytometry analyses of purified circulating EVs identified elevated levels of Calcein-AM/FITC + EVs in PSC compared to healthy controls (Fig. 1C). Cell and tissue origin of serum circulating EVs is heterogenous. For this reason, we identified liver-specific circulating EVs by using two established hepatic markers: the solute carrier family 27 member 5 (SLC27A5) and the asialoglycoprotein receptor 1 (ASGPR1). We identified increased levels of SLC27A5-positive circulating EVs in PSC vs. healthy control while there was no difference in the level of ASGPR1-positive EVs in the two groups (Fig. 1D,E). The discrepancy observed between the two markers may rely on the high specificity of ASGPR1 for hepatocyte and severity of the disease in PSC samples. To further investigate the origin of circulating EVs and to investigate whether isolated circulating EVs carry cholangiocyte markers, we measured the protein concentration of cholangiocyte marker CK19 in circulating EV lysates. We observed a mild but significant increase of EV-encapsulated concentration of CK19 in PSC-derived EVs compared to healthy control-derived EVs (Fig. 1F). These findings were also confirmed by western blot which shows elevated expression of CK19 in circulating PSC-derived EVs compared to control EVs. Despite this difference in CK19 levels, both EV populations expressed the EV markers Alix and CD61 (Fig. 1G). No correlation was identified between total number of circulating EVs and inflammation score. Our findings suggest that a subpopulation of circulating EVs carry hepatic and cholangiocyte-specific markers making them suitable for isolation from serum sample of subjects with PSC.

### Proteome analysis of circulating EVs distinguish healthy from PSC subjects

**Figure 2:**
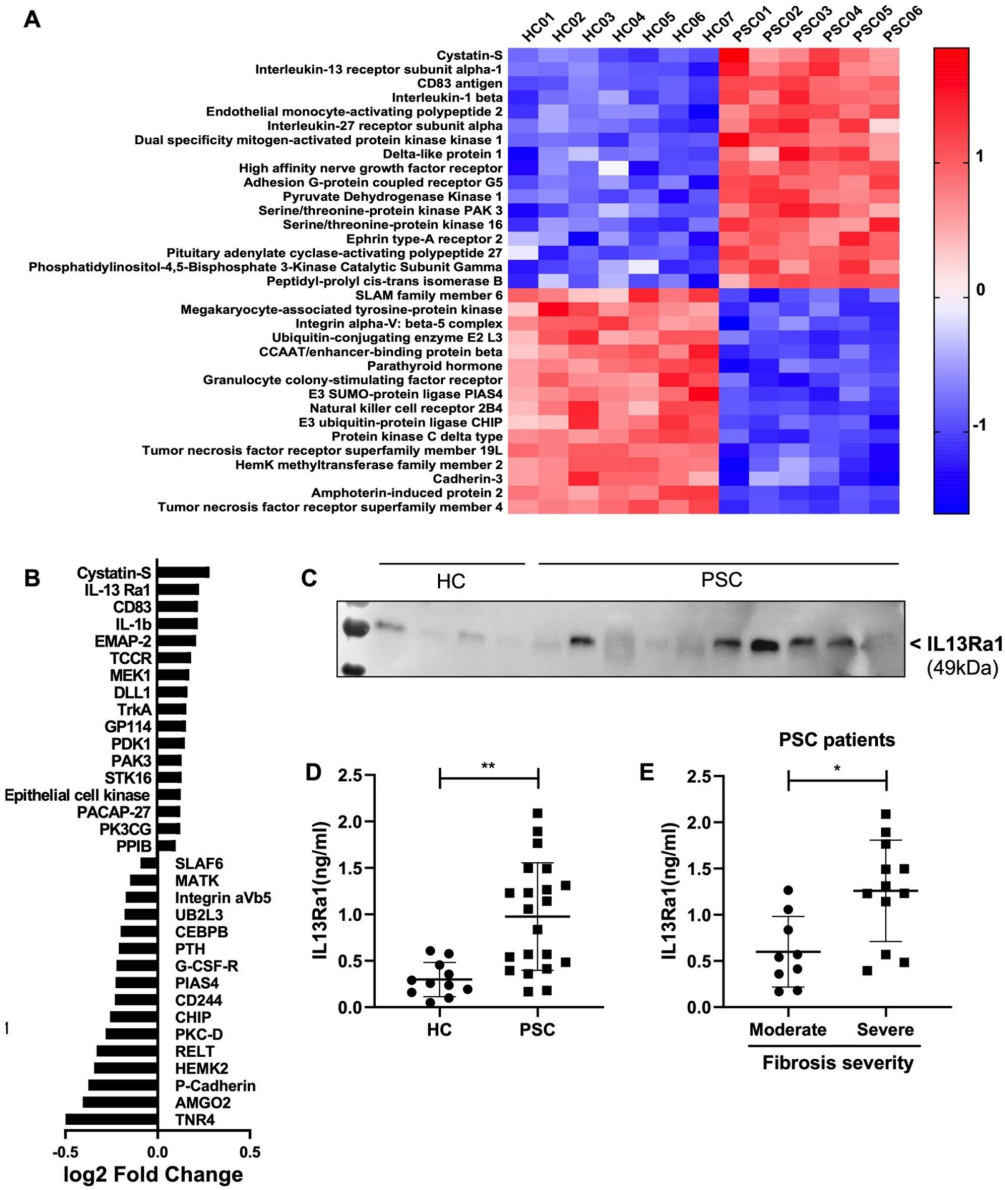
Proteomics analysis of circulating EVs isolated from PSC patients. (**A**) Unsupervised hierarchical clustering analysis of the top 33 differentially expressed circulating EV-proteins in PSC patients (n = 6) vs. healthy controls (n = 7). The top 33 proteins listed on the heatmap were selected based on the adjusted p-value of 0.05 from the PSC vs. healthy controls comparison. Mean and SD of all three groups were used to normalize the expression value. The closer the color is to bright blue, the lower the expression while the closer the color is to bright red, the higher the expression. (**B**) Top 17 UP- and DOWN-regulated proteins in PSC-derived circulating EVs (values expressed as log2 FC). (**C**) Western blot analysis of IL13Ra1 in EVs isolated from healthy controls and PSC patients (a specific band relative to IL13Ra1 is reported in the figure and was cropped from the main gel). (**D**) Identification of IL13Ra1 protein levels by ELISA in EVs isolated from healthy controls and PSC patients. (**E**) Correlation between IL13Ra1 protein levels and fibrosis severity (Ishak score). Kruskal–Wallis test with post-hoc Mann–Whitney test and Bonferroni correction were used for statistical analysis.

A large body of evidence indicates that circulating EVs encapsulate, and shuttle various proteins originally generated in the EV parental cells^24^. Data have also shown that protein composition of circulating EVs mirror the cell wellness and may be tailored to a specific disease, disease stage or response to therapies^4,25^. Despite a recent elegant report highlighting the metabolomic profile of PSC, cholangiocarcinoma and hepatocellular carcinoma subjects^26^, omics studies on PSC-EVs are still scarce and further independent validations are needed. For this reason, we performed a complete proteomics analysis of EVs isolated from healthy controls and PSC subjects. EV protein lysates from both groups were analyzed using the SOMAscan protein array, which contains targets for 1345 unique analytes. A total of 1306 proteins were identified in both healthy control and PSC circulating EVs. Of those, 282 proteins were differentially expressed in PSC compared to healthy control EVs, 130 were up-regulated and 152 were down-regulated in PSC circulating EVs. An unsupervised hierarchical clustering analysis of the top 33 proteins, identified distinct protein profiles in PSC compared to healthy control circulating EVs (Fig. 2A). This indicates that the protein profile of EVs is unique in healthy control compared to PSC subjects. The top upregulated proteins in PSC-EVs are Cystatin-S, IL-13 Ra1, CD83, IL-1β and EMAP-2, which are involved in inflammation, immune response, cell apoptosis, cell senescence, cell motility, endocytosis, differentiation and proliferation, receptor activity and vesicles trafficking. Among the top up-regulated proteins in PSC-EVs, IL13 Ra1 emerged as the most unique marker of PSC-EVs compared to healthy control EVs (Fig. 2B). Western blot-based protein expression analyses revealed an overall greater expression of IL13 Ra1 in PSC-EVs compared to healthy control EVs (Fig. 2C). To confirm the IL13 Ra1 protein expression in EVs, we employed an ELISA-based assay, normally used in diagnostic laboratories as rapid, sensitive, user-friendly and automated diagnostic test. Consistently to our western blot analysis, the ELISA analysis confirmed a significantly elevated level of IL13 Ra1 in PSC-EVs compared to healthy control EVs (Fig. 2D). In addition, our ELISA data also identifies a group of PSC-EVs expressing higher levels of IL13 Ra1 (EV-IL13Ra1^high^) and a second group expressing lower levels of the same protein (EV-IL13Ra1^low^) (Fig. 2D). Based on these findings, we explored whether level of IL13 Ra1 expression in PSC-EVs correlated with disease outcomes. We observed a strong association between EV-IL13 Ra1 expression and severity of liver fibrosis in PSC patients, assessed by Ishak score. Higher levels of IL13 Ra1 in PSC-EVs were associated with severe liver fibrosis while lower levels of EV-IL13 Ra1 were associated to moderate liver fibrosis (Fig. 2E). Our findings suggest that PSC-EVs carry unique protein signatures that may serve as disease-stage biomarkers in PSC subjects.

### Circulating extracellular vesicles carry a unique miRNA signature

**Figure 3:**
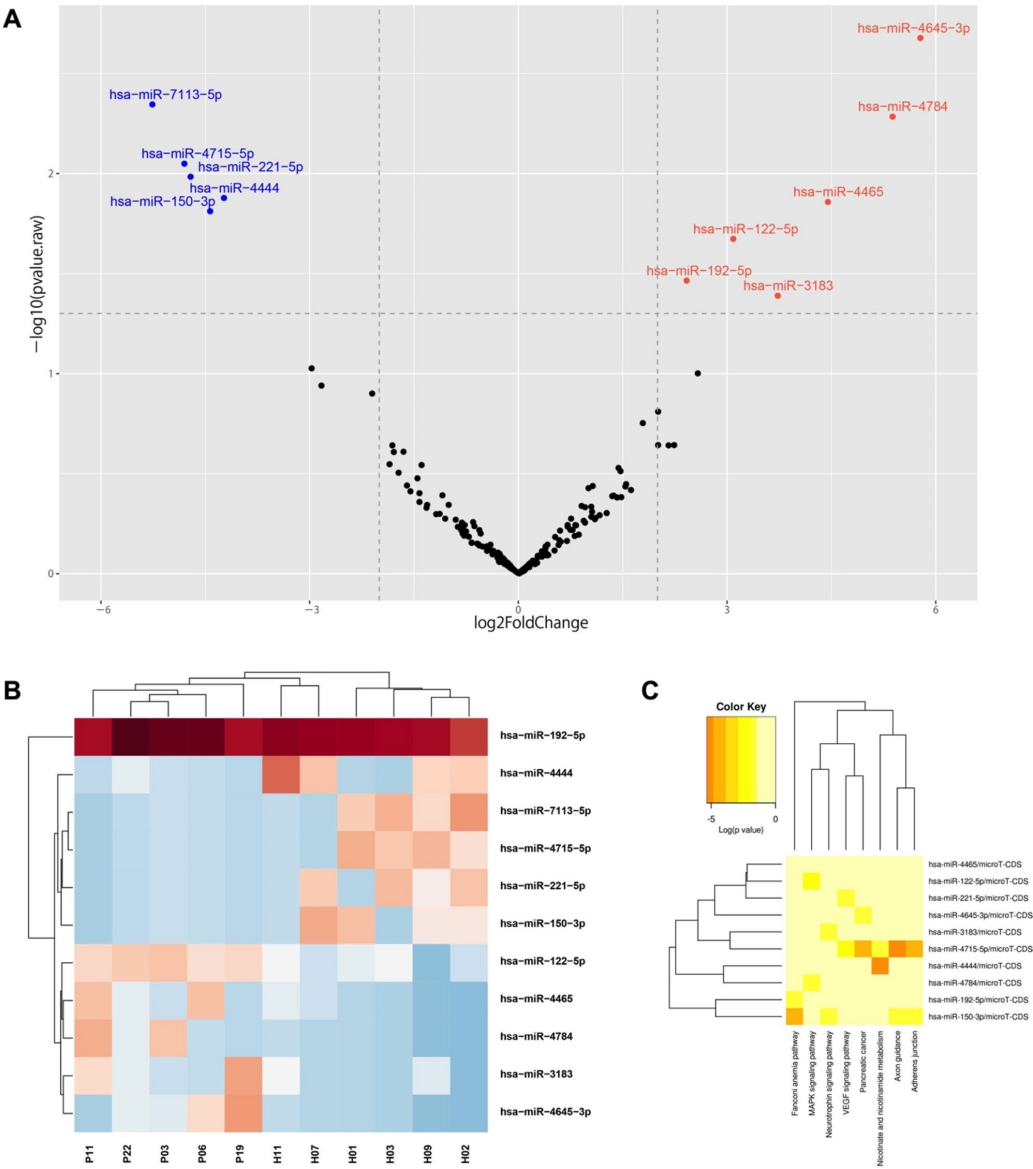
Analysis of miRNA profile in EVs of PSC-derived circulating EVs. (**A**) Volcano plots illustrating significantly differentially abundant EV-miRNAs in PSC patients vs. healthy controls. The − log10 (adjusted p-value) is plotted against the log2 fold-change. (**B**) Unsupervised hierarchical clustering analysis of the top 11 differentially expressed circulating EV-miRNAs identified by miRNA-sequencing in PSC patients vs. healthy controls. An adjusted p-value of 0.05 was used to generate the heatmaps. The closer the color is to bright blue, the lower the expression while the closer the color is to bright red, the higher the expression. (**C**) KEGG pathway enrichment analysis of top 10 differentially expressed EV-miRNAs. KEGG is developed by Kanehisa Laboratories and a copyright request was approved for the use of this tool in this manuscript.

Our proteomics analysis identified various proteins encapsulated in circulating EVs isolated from PSC patients compared to healthy controls. We then explored whether circulating PSC-EVs carried miRNAs, which may be an indication of disease stage, tissue of origin and pathway activated during the progression of the disease. We conducted a whole miRNA-sequencing analysis which identified over 100 different miRNAs, among which eleven were differentially expressed in EVs derived from PSC patients. The top five down-regulated miRNAs identified by our analysis are miR-7113-5p, miR-4715-5p, miR-221-5p, miR-4444; miR-150-3p while the top-upregulated miRNAs are miR-3183, miR-192-5p, miR-122-5p, miR-4465, miR-4784 and miR-4645-3p (Fig. 3A). As many other reports, we identified liver-specific miR-122, which is enriched in circulating EVs released by damaged hepatocytes during chronic liver diseases^27^. The most abundant miRNAs in PSC-derived circulating EVs that we identified was miR-4645-3p. The top eleven differentially expressed miRNAs were included in a hierarchical cluster analysis which identified miR-4444, miR-7113-5p, miR-4715, miR-221, miR-150 particularly up-regulated in healthy controls and miR-122-5p, miR-4465, miR-4784, miR-3183 and miR-4645-3p particularly up-regulated in PSC patients. Despite the miR-192 expression was abundantly up-regulated in both circulating EVs of healthy controls and PSC patients, the latter exhibited a mildly increased expression (Fig. 3B). In order to investigate whether the top 10 differentially expressed miRNAs were associated with a specific pathway, we conducted a pathway enrichment analysis which did not identify any specific pathway association with miR-4645-3p, as the top up-regulated miRNAs in PSC-derived circulating EVs (Fig. 3C). We speculate that the biological and molecular functions of miR-4645-3p have been only partially identified and therefore further studies are needed to elucidate its role in cholangiopathies. Our findings suggest that circulating EVs shuttle several miRNAs that may be combined with the protein signatures in the effort of identifying novel biomarkers for PSC.

### Specific EV-encapsulated miRNAs differentiate PSC patients from healthy controls

**Figure 4:**
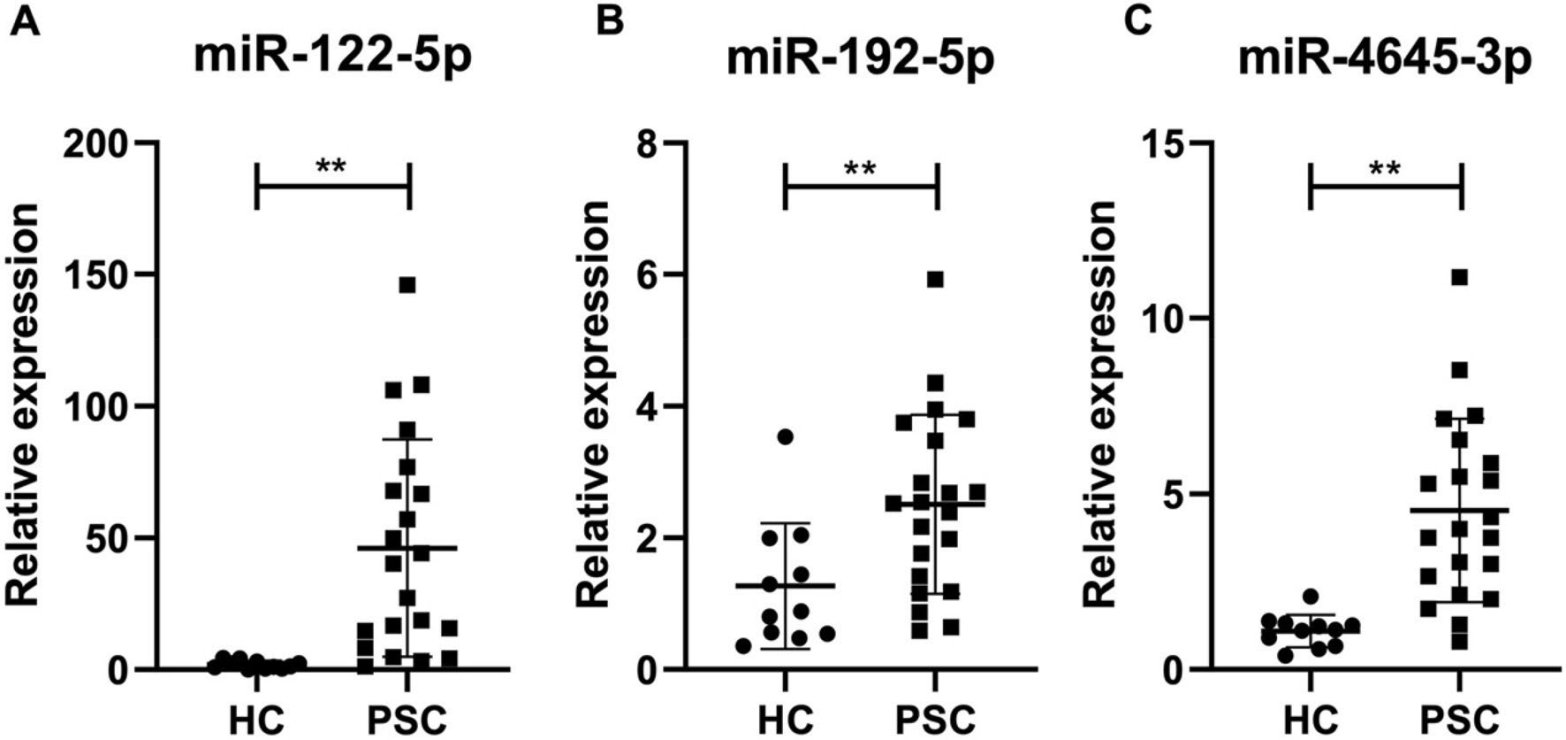
Enrichment of liver-specific miRNAs in PSC-derived EVs. Relative expression of liver-enriched (**A**) miR-122-5p, (**B**) miR-192-5p and (**C**) highly upregulated miR-4645-3p in circulating EVs of healthy controls vs. PSC patients, determined by quantitative PCR. U6 was used as housekeeping miRNA. Values represent mean ± SD. Kruskal–Wallis test with post-hoc Mann–Whitney test and Bonferroni correction were used for statistical analysis.

To validate the findings of miRNA-sequencing and bioinformatics analyses, we determined the expression of three selected miRNAs by quantitative PCR analysis. We measured the relative expression of miR-122-5p, miR-192-5p and our newly discovered and abundantly expressed miR-4645-3p in circulating EVs from healthy control and PSC patients. Our findings aligned with previous reports which showed hepatic expression of miR-122 and miR-192 in healthy livers while described enrichment of these two miRNAs in circulating EVs derived from damaged hepatocytes. The increased expression of these miRNAs in circulating EVs during chronic liver diseases was associated to an equally significant decrease in tissue^27^. In accordance with these reports, we also observed low levels of miR-122-5p in circulating EVs of healthy controls while circulating EVs of PSC patients contained significantly elevated levels of this miRNA (Fig. 4A). Similarly, to miR-122-5p, also the expression of miR-192-5p was greater in circulating PSC-EVs compared to healthy controls (Fig. 4B). Notably, the expression of our newly identified miR-4645-3p was significantly up-regulated in circulating PSC-EVs compared to healthy controls (Fig. 4C), suggesting that this miRNA may play a crucial role in both the development or as biomarker of PSC.

## Discussion

At present, no reliable biomarkers have been identified that can predict the progression of primary sclerosing cholangitis and that can be used as surrogate diagnostic and/or prognostic biomarkers in patients with PSC^2^. Prior reports have identified circulating small extracellular vesicles that carry various potential protein-based biomarkers for PSC^4^. In this study we report that a well-characterized cohort of patients with histologically confirmed primary sclerosing cholangitis exhibit greater levels of circulating large extracellular vesicles compared to control subjects. Our report confirms the findings of previous studies which identified circulating EVs in PSC patients and provides additional novel data on rather larger circulating EVs—which seem more abundant than smaller EVs in patients with PSC—it identifies the EV protein and miRNA cargo and further validate the potential clinical relevance of one of the most abundant EV-encapsulated protein and miRNA as biomarker candidate for PSC patients. Over the past decade, circulating extracellular vesicles have been studied in various biological fluids with the goal to validate them as reliable, noninvasive and inexpensive biomarker candidates for various human diseases^22,28,29^. However, only a handful number of studies have reported EV dynamics, protein and miRNA cargo and performance in PSC patients. In this study, we isolated circulating EVs from serum samples of biopsy-proven cohort of PSC patients and corresponding control healthy subjects and we performed a complete morphological and physical characterization as well as multi-omics unbiased analyses of EV cargo. Isolation and purification of circulating EVs were performed according to MISEV2018 guidelines by using differential centrifugation and size exclusion chromatography (SEC)^23,30,31^. Combining differential centrifugation followed by SEC minimizes potential contamination of non-EV circulating components, such as serum proteins, lipoproteins, and other contaminants. A first determination of EV size and amount revealed that our population was composed mainly of large EVs and that EV levels were greater in PSC patients compared to control healthy subjects. Compared to other reports, our study identified a significant difference in EV concentration between PSC patients and control healthy subjects, likely due to the fact that we focused on larger EVs (e.g. microvesicles), compared to previous studies that focused on smaller vesicles (e.g. exosomes). We postulate that the larger size of our EV population may derive from the different EV isolation and identification methods used or may indicate that larger EVs are more abundantly released in circulation of patients with chronic liver diseases as a consequence of hepatocyte or cholangiocyte cell death. In support of this, we identified not only common markers of EVs but also hepatocyte and cholangiocyte-specific markers such as ASGPR1, SLC27A5 and CK19. Strikingly, our unbiased proteomics analyses identified a distinct protein signature in large circulating PSC-EVs compared to healthy control EVs. As compared to previous reports on PSC-EVs, our proteomics analysis identified different proteins enriched in PSC-EVs, likely due to the different subpopulation of EVs isolated and analyzed in our study. Indeed, our differential centrifugation and size-exclusion chromatography (SEC) procedures allow for enrichment of EVs > 200 nm, which is the lowest size detectable by the standard FACS sorting machine used in our study for EV quantification. Pathway enrichment analyses indicate that the highest expressed proteins in PSC-EVs belong to the inflammatory/cytokine, cell adhesion and migration pathways, confirming previous reports and mirroring common PSC outcomes such as inflammation and wound-healing. The top inflammatory proteins highly expressed in PSC-derived EVs compared to healthy control subjects were IL-1β, IL-13 Ra1, CD83 and IL27ra while the most abundant protein involved in TGF-β signaling and cell migration were EMAP-2, MEK-1 and DLL1. Among all our validation follow-up analyses on the top expressed proteins in PSC-derived EVs, the IL-13 receptor IL-13Ra1 surged as the most robust and highly expressed protein in PSC-derived EV lysates, compared to healthy control subjects. IL-13 has been increasingly recognized for its role in inflammatory conditions, such as asthma, ulcerative colitis and eosinophilic oesophagitis but also in diseases with a strong fibrosis component. When IL-13 binds to its receptor IL-13Ra1, it transduces the intracellular signaling pathway that triggers the activation of STAT6, PI3K and MAPK which contribute to various downstream cell responses including cell motility, adhesion and response to stress^32^. IL-13Ra1 was not only elevated in PSC-derived EV protein lysates but also correlated with fibrosis severity in PSC patients, suggesting that this receptor may represent a potential surrogate biomarker for PSC and biliary fibrosis. In addition to the proteome analysis of circulating EVs, our report provides novel insights on the miRNA composition of EVs isolated from PSC patients or control subjects. miRNAs encapsulated in circulating EVs have been identified and characterized in several human diseases and conditions, including cancer^33^, alcohol-associated liver disease^34^, kidney disease^35^, pulmonary disease^36^. However, the role of miRNAs and particularly of those encapsulated in circulating EVs as potential biomarkers in PSC patients is still incompletely understood. In this study, we explored the miRNA cargo of PSC-derived circulating EVs compared to that of circulating EVs isolated from control subjects. Several independent studies have identified miR-122-5p and miR-192-5p differentially up-regulated and specifically enriched in circulating EVs isolated from patients with chronic liver diseases^34,37^. Accordingly, circulating extracellular vesicles of PSC patients exhibited up-regulation of both miRNAs compared to EVs of control subjects. These findings support previous evidence that during liver injury and hepatic cell death miRNAs are specifically encapsulated in EVs and shuttled in the blood stream overtime during disease progression. In addition to previously described miRNAs, we identified and characterized miR-4645-3p, a novel miRNA particularly enriched in PSC-derived EVs and that correlated with levels of miR-122-5p and miR-192-5p. Limited data is currently available on miR-4645-3p and points to a role of this miRNA in age-related disorders and ER stress, which may be indicative of the significant senescence occurring in PSC. In summary, this report provides evidence that PSC patients are enriched particularly of large EVs that contain specific protein signatures compared to control subjects. In addition, we identified specific EV-encapsulated protein and miRNA that may open new opportunities for the development of non-invasive diagnostic tools for PSC patients. Enrichment and encapsulation of specific bioactive molecules in circulating EVs may constitute an advantage for long-term preservation of miRNAs and proteins in circulation and may improve sensitivity of current detection methods.

## Study limitations

A limitation of this study includes the relatively low number of samples analyzed and lack of a disease-control cohort. Therefore, future validation studies using larger number of patient samples and disease-control cohorts are needed to confirm the specificity of EV-protein signatures identified in our study as biomarkers of PSC. Future studies to assess the disease-specificity of the EV markers uncovered are warranted and will require the inclusion of patients with advanced liver disease from various etiologies.

## Supplementary Information

Supplementary Figure 1.

## Abbreviations

BMI: Body mass index
ALT: Alanine aminotransferase
GGT: Gamma-glutamyl transferase
INR: International normalized ratio
MELD: Model for end-stage liver disease
ELF: Enhanced liver fibrosis
NAFLD: Non-alcoholic fatty liver disease
NAS: NAFLD activity score
HVPG: Hepatic venous pressure gradient
CSPH: Clinically significant portal hypertension
