# Supplementary figures and images for: Protein and miRNA profile of circulating extracellular vesicles in patients with primary sclerosing cholangitis
Source: Sci Rep. 2022 Feb 22;12:3027. doi: 10.1038/s41598-022-06809-0 (PMC8863778; doi:10.1038/s41598-022-06809-0)

**A**

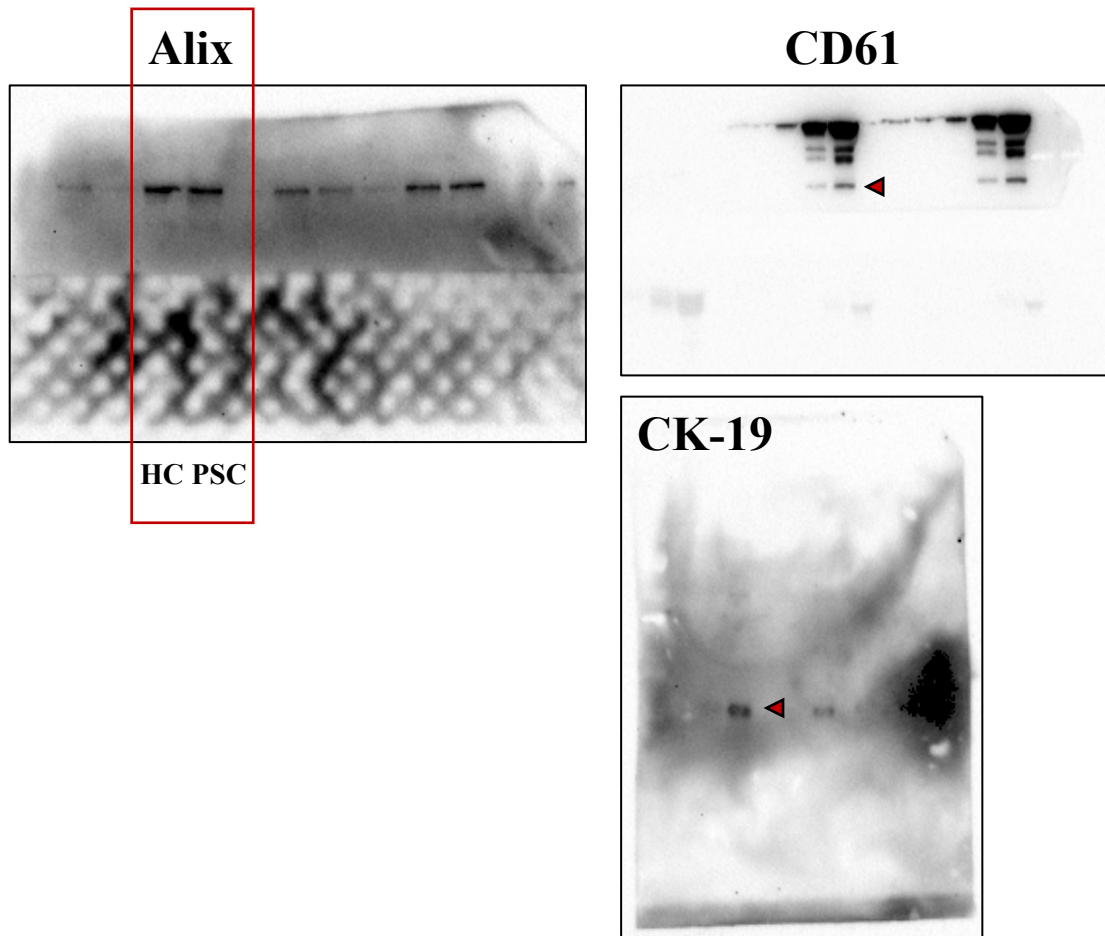

**B**

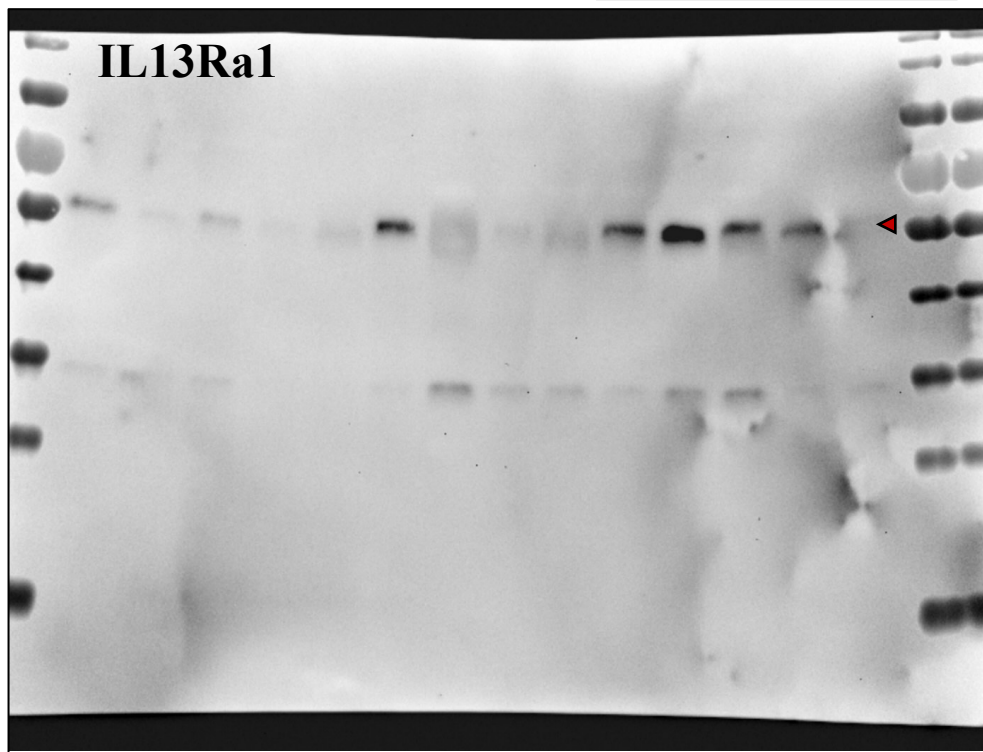

**Supplemental Figure 1.** Original uncropped western blots for (A) Alix, CD61, CK-19 and (B) IL13Ra1.

Supplement: Supplementary file 1 — Supplementary Figure 1. [file 41598_2022_6809_MOESM1_ESM.pdf]
